# Myosin X regulates neuronal radial migration through interacting with *N*-cadherin

**DOI:** 10.3389/fncel.2015.00326

**Published:** 2015-08-18

**Authors:** Mingming Lai, Ye Guo, Jun Ma, Huali Yu, Dongdong Zhao, Wenqiang Fan, Xingda Ju, Muhammad A. Sheikh, Yousra S. Malik, Wencheng Xiong, Weixiang Guo, Xiaojuan Zhu

**Affiliations:** ^1^Key Laboratory of Molecular Epigenetics, Ministry of Education and Institute of Cytology and Genetics, Northeast Normal UniversityChangchun, China; ^2^Department of Biochemistry and Molecular Biology, School of Basic Medical Sciences, Dali UniversityDali, China; ^3^Department of Neurology, Georgia Regents University, AugustaGA, USA; ^4^State Key Laboratory for Molecular and Developmental Biology, Institute of Genetics and Developmental Biology, Chinese Academy of SciencesBeijing, China

**Keywords:** Myo10, *N*-cadherin, interaction, neuronal migration, cell adhesion, membrane trafficking

## Abstract

Proper brain function depends on correct neuronal migration during development, which is known to be regulated by cytoskeletal dynamics and cell-cell adhesion. Myosin X (Myo10), an uncharacteristic member of the myosin family, is an important regulator of cytoskeleton that modulates cell motilities in many different cellular contexts. We previously reported that Myo10 was required for neuronal migration in the developing cerebral cortex, but the underlying mechanism was still largely unknown. Here, we found that knockdown of Myo10 expression disturbed the adherence of migrating neurons to radial glial fibers through abolishing surface Neuronal cadherin (*N*-cadherin) expression, thereby impaired neuronal migration in the developmental cortex. Next, we found Myo10 interacted with *N*-cadherin cellular domain through its FERM domain. Furthermore, we found knockdown of Myo10 disrupted *N*-cadherin subcellular distribution and led to localization of *N*-cadherin into Golgi apparatus and endosomal sorting vesicle. Taking together, these results reveal a novel mechanism of Myo10 interacting with *N*-cadherin and regulating its cell-surface expression, which is required for neuronal adhesion and migration.

## Introduction

During neocortical brain development, postmitotic neurons newly generated in the ventricular zone (VZ) and subventricular zone (SVZ) migrate radially toward the pial surface to form the cortical structure (Nadarajah et al., 2001; Gupta et al., 2002; Kriegstein and Noctor, 2004). Neuronal migration comprises several steps with the following morphological changes: firstly, neurons display a multipolar morphology in the lower part of the intermediate zone (IZ; Tabata et al., 2009), and subsequently transform into bipolar-shaped locomotion neurons in the upper part of the IZ. Then the neurons migrate over a long distance along radial glial fibers from the IZ to arrive close to the top of the cortical plate (CP). At the final phase, the locomotion neurons change their migration mode into the terminal translocation mode (Hatanaka and Murakami, 2002; Nadarajah and Parnavelas, 2002; Tabata and Nakajima, 2003). Among the migration, the locomotion mode is the main contributor to neuronal migration and cortical layer formation, which depends on the interaction between migrating neurons and radial glial cells (RGCs). However, the precise molecules that mediate the interaction between migrating neurons and RGCs remain largely unknown.

Neuronal cadherin (*N*-cadherin), a transmembrane cell adhesion molecule which is widely expressed in developmental and mature brain tissues. It is required to maintain tissue integrity, as well as regulate cell polarity and glial-guided radial migration (Gumbiner, 1996; Shikanai et al., 2011; Gartner et al., 2012). As a homophilic adhesion molecule, *N*-cadherin regulates cell–cell interaction by the extracellular domains in a calcium-dependent way (Takeichi, 1995). The cytosolic domain of *N*-cadherin is associated with the actin cytoskeleton through β- and α-catenins to modulate the cell adhesion and motility (Yap et al., 1997; Gumbiner, 2005). It has been reported that *N*-cadherin is expressed in both migrating neurons and radial glial fibers and plays roles in a variety of processes during the cortical development (Takeichi, 1995; Kadowaki et al., 2007; Zhang et al., 2009; Kawauchi et al., 2010; Franco et al., 2011; Jossin and Cooper, 2011; Shikanai et al., 2011; Gartner et al., 2012). *N*-cadherin and its effector, β-catenin, are expressed in radial glia cells, where they regulate their proliferation and neurogenesis (Brault et al., 2001; Machon et al., 2003; Woodhead et al., 2006; Kadowaki et al., 2007; Zhang et al., 2009; Mutch et al., 2010). Recent findings suggest additional functions of *N*-cadherin in glial-independent somatic translocation, and glial-guided migration (Kawauchi et al., 2010; Franco et al., 2011; Jossin and Cooper, 2011). The diverse functions of *N*-cadherin in the neocortex indicate that its activity must be tightly controlled. However, it is still not clear how exactly formation of *N*-cadherin is regulated.

Myosin X (Myo10), an important regulator of cytoskeleton remodeling, is critical for filopodia formation as well as cell motility (Berg and Cheney, 2002; Kerber et al., 2009; Wang et al., 2009; Watanabe et al., 2010). As a motor protein, it shares conserved structural features at the N-terminal domain that binds with actin filaments (Nagy et al., 2008; Sun et al., 2011). The C-terminal end of the tail domain has been reported to transport proteins including Mena/VASP, β-integrin, DCC, ALK6 and VE-cadherin (Tokuo and Ikebe, 2004; Zhang et al., 2004; Pi et al., 2007; Zhu et al., 2007; Almagro et al., 2010). Meanwhile, Myo10 is required for migration of endothelial cells, polarized epithelial cells, cranial neural crest cell, as well as neurons during the brain development (Hwang et al., 2009; Nie et al., 2009; Almagro et al., 2010; Liu et al., 2012). Perturbing of Myo10 function inhibits neuronal polarization and transition from multipolar to bipolar shape (Raines et al., 2012; Yu et al., 2012; Ju et al., 2013). Despite of the importance of Myo10 in cytoskeleton dynamics and cell motility, the detailed functions of Myo10 in the development of neocortex remain to be elucidated.

In this study, we provided evidence that Myo10 interacted with *N*-cadherin to regulate neuronal radial migration. Using an acute loss-of-function approach with RNA interference, we demonstrated that Myo10 contributed to the tight interaction between migrating neurons and radial glial fibers, which is essential for neuron migration during cerebral cortex development. Surprisingly, *N*-cadherin could rescue the defects caused by knockdown of Myo10. Furthermore, we found that Myo10 formed a complex with *N*-cadherin, and then regulated *N*-cadherin surface expression through controlling its membrane trafficking. Taken together, these results illustrate the critical role of Myo10 in the *N*-cadherin-dependent neuronal radial migration.

## Materials and Methods

### Antibodies and Plasmids

Primary antibodies were rabbit anti-Myo10 (Zhu et al., 2007), mouse anti-*N*-cadherin (BD Biosciences); rabbit anti-*N*-cadherin (Sigma-Aldrich; Santa Cruz, CA, USA), mouse anti-EGFP (Invitrogen; Santa Cruz, CA, USA), rabbit anti-EGFP (Invitrogen), rabbit anti-RFP (Abcam), mouse anti-β-catenin (Santa Cruz, CA, USA), mouse anti-β-actin (Abcam), mouse anti-Tuj1 (Abcam), mouse anti-Nestin (Abcam), mouse anti-MAP2 (Sigma-Aldrich), mouse anti-GM130 (BD, Bioscience), mouse anti-sytaxin6 (BD, Bioscience), mouse anti-EEA-1 (BD, Bioscience), rabbit anti-Bip (Santa Cruz, CA, USA), rabbit anti-Rab7 (Santa Cruz, CA, USA), rabbit anti-Rab11, Digitonin (Sigma-Aldrich). Myo10 small hairpin RNA (shRNA) targeting regions of 5′-GCAGCTGATCCAAGATATT-3′ together with enhanced green fluorescent protein (EGFP) was prepared as previously described (Zhu et al., 2007). To construct Ncad shRNA, oligonucleotides targeting two distinct regions in the *N*-cadherin coding sequence (Ncad shRNA: 5′-GTGCAACAGTATACGTTAATA-3′) (Kawauchi et al., 2010) were inserted into pSuper vector. None-silencing shRNA was a control containing no homology to known mammalian genes. The plasmid of mCherry-Myo10 was kindly provided by Dr. Staffan Strömblad (Karolinska Institutet, Sweden). The cDNA encoding *N*-cadherin was amplified by PCR and subcloned into mammalian expression vector pDsRed2-C1. The cDNA sequences corresponding to the cytoplasmic domain of *N*-cadherin were cloned into the pGEX-5x-1 vector for GST fusion protein.

### *In Utero* Electroporation

Pregnant mice were anesthetized and their uterine horns were exposed with a midline laparotomy incision. Embryos were removed and carefully placed on humidified gauze pads. Plasmid DNA plus 0.01% Fast Green (Fluka) was injected into the lateral ventricles of the embryonic brain with a glass micropipette. A volume of 2 μl of shRNA plasmids (2 μg/μl) or expression constructs (2 μg/μl) were co-injected with the cytomegalovirus (CMV) early enhancer element and chicken β-actin (CAG) promoter-EGFP-expressing plasmids. For rescue experiments, expression constructs were co-injected with shRNA and CAG-EGFP plasmids. For electroporation, 5 × 50 ms, 37 V square pulses separated by 950 ms intervals were delivered with forceps-type electrodes connected to an ECM 830 electroporator (BTX Harvard Apparatus). The uterus was then replaced into the abdominal cavity, and the abdomen wall and skin were sutured using the surgical needle and thread. The whole procedure was completed within 40 min. The pregnant mouse was warmed in an incubator until it became conscious, and embryos were allowed developed *in utero* for the time indicated.

### *N*-Cadherin Binding Assay

Binding of neurons to *N*-cadherin extracellular domain-Fc (NcadECD-Fc) fusion protein (mouse) (R&D Systerms # 6626-NC-050) was done as previously described (Qin et al., 2005). NcadECD-Fc was diluted in Hank’s balanced salt solution (HBSS) with 1 mM CaCl_2_ and aliquots of 50 μg ml^-1^ were stored at -80°C in tubes blocked with 3% BSA (wt/vol). Ninety six well high binding plates (JET BIOFIL, FEP-101-896) were coated with 12.5 μg ml^-1^ NcadECD-Fc or 20 μg ml^-1^ poly-L-lysine (PLL) (Sigma), overnight at 4°C. Wells were then blocked with 3% BSA for 2 h at 25°C, then after washed three times with HBSS + 1.2 mM CaCl_2_. Neuron suspensions were prepared from embryonic day 16 (E16) mouse embryo telencephalons. About 10^5^ cells were added to each well and cultured overnight in DMEM-F12 medium with 2% B27 and 1 × penicillin and streptomycin. Next day, cells bound to plates were imaged and counted. After gentle washing with HBSS + 1.2 mM CaCl_2_ cells were imaged and counted again. The data were presented as the percentage of cells remaining in each washed well, compared with the unwashed control.

### Cortical Neuron Culture and *In Vitro* Electroporation

Cortical neuron cultures were prepared as described previously (Zhu et al., 2007). In brief, E16 mouse embryonic cerebral cortices were digested with 0.125% trypsin at 37°C for 25 min and dissociated by pipetting in DMEM/F12 with 10% fetal bovine serum (FBS). Electroporation was performed using Amaxa mouse neuron nucleofector Kit (Amaxa Biosystems) according to the manufacturer’s instructions. About 2–2.5 × 10^6^ neurons were resuspended in 100 μl of Nucleofectamine solution containing 3 μg of plasmid and plated with 1.5 × 10^5^ per cover slip coated with 1 mg/ml PLL. After 4–5 h the medium was changed to Neurobasal medium (Invitrogen) with 2% B27 supplement (Gibco) and 2 mM L-glutamine (Sigma).

### Time-Lapse Imaging in Cortical Slice

*In utero* electroporation was performed as described above at E15.5. Two days after electroporation, embryonic brains were dissected out in cold artificial cerebrospinal fluid. Brain slices (300 μm thick) were sectioned with the Leica Vibratome VT1000. To visualize neuronal migration, slices were transferred onto Millicell inserts (Millipore) in Neurobasal medium (Invitrogen) containing 2% B-27 supplement, 2 mM L-glutamine and penicillin/streptomycin (50 U/50 μg/ml). The glass-bottomed dish was then fitted into a temperature-controlled chamber on the microscope stage for 15 h at 37°C under 5% CO_2_ air atmosphere. Live cell imaging was done using Olympus FV1000 Viewer laser scanning confocal microscope.

### Immunofluorescence

Cells were fixed with 4% paraformaldehyde (PFA) in phosphate buffered saline (PBS) for 15 min at room temperature, permeabilized with 0.15% Triton X-100 for 10 min and blocked in 2% BSA for 1 h in PBS. Subsequently, cells were incubated with primary antibodies overnight at 4°C and washed five times with 0.1% Tween-20 in PBS. And they were incubated with appropriate fluorochrome-conjugated secondary antibodies for 1 h. Cells were washed five times and cover-slips with 75% glycerol. Images were captured using Olympus FV1000 View confocal microscope (Tykyo, Japan) and Zeiss LSM 780 confocol microscope (Carl Zeiss, German).

For tissues, embryos mice were fixed in 4% PFA in PBS overnight at 4°C, cryoprotected in 30% sucrose containing PBS. Brains were frozen in optimal cutting temperature compound (OCT) and cut on a freezing microtome into 20-μm-thick coronal sections. Frozen sections were washed with PBS, and antigen-retrieved by immersion of the slides in 0.01 M sodium citrate buffer, pH 6.0 at 95°C for 5 min. Sections were then blocked with 2% BSA in 0.2% Triton X-100/PBS for 1 h, followed by immunostaining as above.

We modified an immunofluorescence method developed by Jiajia Liu for the endosome markers (Fu et al., 2011). Cells were rinsed with KHM buffer (20 mM HEPES-pH 7.4, 110 mM potassium acetate, and 2 mM magnesium acetate) and then treated with KHM buffer containing 25 μg⋅ml^-1^digitonin for 5 min on ice. Cells were rinsed once with KHM buffer and fixed with 4% PFA in PBS for 10 min at room temperature. Cells were blocked with PBS containing 5% BSA and 0.5% Tween-20 for 15 min followed by overnight incubation at 4°Cwith primary antibody. Appropriate secondary antibodies conjugated with Alexa Fluor 405, Alexa Fluor 488, or Alexa Fluor 546 were used for detection.

### Immunoprecipitation

Immunoprecipitation was carried out as previously described. Briefly, cell lysates (500 μg protein) were incubated with the indicated antibodies (1–2 μg) at 4°C overnight in a final volume of 1 ml modified RIPA lysis buffer with protease inhibitors. After the addition of protein A-G-agarose beads, the reactions were incubated at 4°C for 2 h. The immune-precipitate complexes were collected by centrifugation and washed three times with washing buffer (20 mM Tris-HCl, 10 mM NaCl, 1 mM EDTA, 0.5% NP40). The agarose beads were resuspended in 30 μl of 2 × loading buffer and boiled for 5 min to release the protein. After 1 min of centrifugation, the supernatants were separated by SDS-polyacrylamide-gel electrophoresis (SDS-PAGE). The proteins were transferred to a poly vinylidenedifluoride (PVDF) membrane, probed with the listed antibodies and developed with Amersham^TM^ ECL^TM^ Prime Western Blotting Detection Reagent (GE Healthcare).

### GST-Pulldown

Expression vector containing GST-Ncad cytoplasmic domain (GST-Ncad-CD) fusion protein was constructed by ligation into the *Bam*H I and *Eco*R I sites of pGEX-5x-1. GST-Ncad-CD fusion protein was purified with glutathione-Sepharose^TM^ 4B beads (GE Healthcare) in accordance with manufacturer’s protocol. About 500 μg of cell lysate from HEK293T cells transfected with indicated plasmids were incubated with 2–5 μg of GST-Ncad-CD fusion protein in RIPA buffer at 4°C over night. Glutathione-Sepharose^TM^ 4B beads were used to capture the GST-Ncad-CD fusion protein with the interacting proteins. The bound proteins were released by heat denaturing into protein loading buffer. Samples were then resolved by SDS-PAGE and subjected to immunoblotting.

### Imaging and Quantification

Images were acquired using Olympus FV1000 View confocal microscope with FV10-ASW 1.7 software, or Zeiss LSM 780 confocol microscope with FV10-ASW 1.7 software, where only the brightness, contrast and color balance were optimized. The analysis of cell numbers and the length between neurons and radial glial fibers were performed with ImageJ software. For quantification of radial migration layers were drawn following nuclear staining and EGFP-expressing cells were counted using ImageJ software. Multipolar cells were defined as cells with more than three projections. The non-radially migrating neurons were defined as the neurons with their leading processes deviating from the normal radial trajectory by more than 45°, measured with ImageJ software. Tests of normality were carried out by Shapiro–Wilk test. Only the data with normal distribution (*p* > 0.05) was used for statistical analysis. Statistical analysis was performed using unpaired two-tailed student’s *t*-test. The data were presented as mean ± SEM.

## Results

### Myo10 Regulates *N*-Cadherin-Mediated Neuronal Adhesion

**FIGURE 1:**
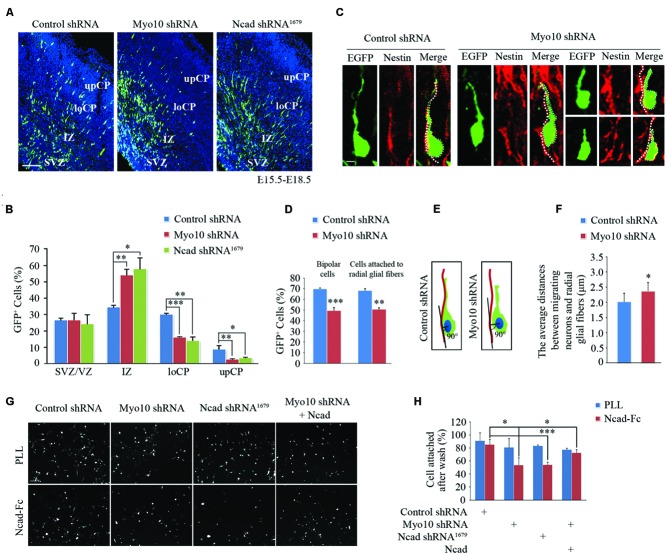
**Knockdown of Myo10 impaired *N*-cadherin-mediated neuronal adhesion. (A)** The representative images of E18.5 embryonic cerebral cortex electroporated with indicated plasmids at E15.5 were immunostained with anti-EGFP antibody (green). Scale bar, 100 μm. **(B)** Quantification of the relative distribution (percentage) of EGFP-expressing cells in the SVZ/VZ, IZ, loCP, and upCP, respectively. Histograms show mean ± SEM from at least three brains. **(C)** Representative images of EGFP-expressing cells and nestin-positive radial glial fibers after *in utero* electroporation with indicated plasmids. Scale bar, 20 μm. **(D)** Quantification of the percentage of bipolar EGFP-expressing cells and that were in contact with the nestin-positive radial glial fiber in upIZ and loCP regions. Histograms show mean ± SEM from at least three brains. **(E)** Schematic diagram of quantitative analysis. **(F)** Quantification of the average distance between the center of the soma of the cells with one leading process and the nestin-positive radial glial fiber. Histograms show mean ± SEM from at least 30 cells. **(G)** The representative images of EGFP-expressing neurons attached to NcadECD-Fc or PLL coated plate. Scale bar, 100 μm. **(H)** Quantification of the percentage of attached EGFP-expressing cells. Histograms show mean ± SEM from at least three independent experiments. ^∗^*p* < 0.05; ^∗∗^*p* < 0.01; ^∗∗∗^*p* < 0.001.

In the developing cortex, neurons have been known to attach to and migrate along the radial glial fibers and fulfill cortex layer patterning. Disruption of these processes leads to abnormal neuronal migration and cortex development. Using inter uterus electroporation, we found disturbance of Myo10 in newborn neurons led to impaired radial migration, with an accumulation of EGFP^+^ cells in IZ and a decrease of EGFP^+^ cells in CP (**Figures 1A,B**), which is consistent with our previous studies (Yu et al., 2012; Ju et al., 2013). To characterize more closely the migration defects caused by Myo10 knock-down, we analyzed the neurons detained in the upIZ and loCP regions. Knockdown of Myo10 led to the decreases of EGFP^+^ bipolar cells [Control shRNA: 70.09 ± 0.72%, *n* = 4; Myo10 shRNA: 49.59 ± 2.77%, *n* = 4; *t*-test, ^∗∗∗^*p* < 0.001] and such cells that adhered to Nestin positive fiber [Control shRNA: 68.41 ± 1.68%, *n* = 4; Myo10 shRNA: 50.74 ± 1.47%, n = 4; *t*-test, ^∗∗^*p* = 0.003] (**Figures 1C,D**). The Myo10-knockdown neurons did not tightly attach to the radial glial fibers with a short or irregular leading process compared to control neurons (**Figure 1C**). Next, the vertical length between the center of the soma of EGFP^+^ bipolar neurons and the Nestin^+^ radial glial fibers was measured to indicate the neuronal adhesive characteristics (Shikanai et al., 2011; **Figure 1E**). Although loss of Myo10 has no effect on the radial glial fiber formation (Ju et al., 2013), Myo10 deficiency led to an increase of the vertical length between the center of the soma of bipolar neurons and the radial glial fibers, comparing to control cells [Control shRNA: 2.01 ± 0.28 μm, *n* = 32; Myo10 shRNA: 2.36 ± 0.29 μm, *n* = 35; *t*-test, ^∗^*p* = 0.023] (**Figure 1F**). Thus, above data suggested Myo10 might involve in the regulation of cell–cell interaction between the locomoting neurons and radial glial fibers.

The abnormally loose adherence of Myo10-deficient migrating neurons with their parental RGCs might be caused by defect of cell adhesion molecules that mediate these interactions. Recent studies have shown that *N*-cadherin-mediated adhesion is required for glial-guided migration (Shikanai et al., 2011). Interestingly, we found respectively disrupting Myo10 or *N*-cadherin function resulted in similar neuronal migration defects (**Figures 1A,B**). To test whether Myo10 modulates the functional *N*-cadherin on the cell surface, we isolated mouse E16.5 cortical neurons and cultured onto poly-L-lysine (PLL, non-*N*-cadherin extracellular matrix) or *N*-cadherin extracellular domain-Fc (NcadECD-Fc) coated plates. We found neurons transfected with control shRNA, Myo10 shRNA or *N*-cadherin shRNA had similar adherent ability to PLL (**Figures 1G,H**). However, comparing to control neurons, we found the neurons transfected with Myo10 shNRA or *N*-cadherin shRNA showed equal lower adherent ability to NcadECD-Fc [Control shRNA: 85.48 ± 7.90%, n = 5; Myo10 shRNA: 53.95 ± 11.38%, n = 5; *t* test, ^∗^*p* = 0.021; Ncad shRNA^1679^: 54.30 ± 4.12%, n = 5; *t*-test, ^∗^*p* = 0.01] (**Figures 1G,H**). Furthermore, we found overexpressing full-length *N*-cadherin (RFP-Ncad) in Myo10-knockdown neurons could rescue the adherent ability onto NcadECD-Fc [Myo10 shRNA + RFP-Ncad: 72.98 ± 4.89%, *n* = 5; *t*-test, ^∗∗^*p* = 0.009] (**Figures 1G,H**). These results suggested that Myo10 regulated *N*-cadherin-mediated neuronal adhesion.

### Overexpression of *N*-Cadherin Rescues the Defect of Cortical Neuronal Migration Caused by Loss of Myo10

**FIGURE 2:**
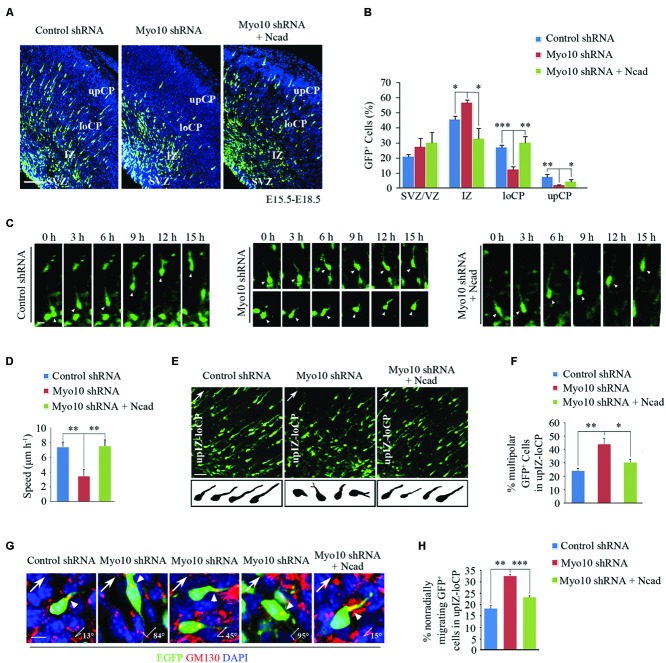
**Overexpression of *N*-cadherin rescued the neuronal migration defect by knockdown of Myo10. (A)** The representative images of E18.5 embryonic cerebral cortex electroporated with indicated plasmids at E15.5 were immunostained with anti-EGFP antibody (green). Scale bar, 100 μm. **(B)** Quantification of the relative distribution (percentage) of EGFP-expressing cells in the VZ/SVZ, IZ, loCP and upCP for each condition. Histograms show mean ± SEM from at least three brains. **(C)** The representative images of time-lapse observation of migrating neurons in cultured cortical tissues after electroporating with indicated plasmids. White arrowheads indicates the EGFP-positive migrating neurons. Scale bar, 5 μm. **(D)** Quantification of the relative speed of EGFP-expressing neurons. Histograms show mean ± SEM from at least 20 cells. **(E)** Microscope images of EGFP-expressing neurons in upIZ and loCP regions of E18.5 mouse cortex (upper panel) and drawings illustrate the morphology of EGFP-expressing neurons for each condition (lower panel). Scale bar, 20 μm. **(F)** Quantification of the percentage of multipolar EGFP-expressing cells in upIZ and loCP regions. Histograms show mean ± SEM from at least three brains. **(G)** Representative examples of EGFP-expressing migrating cells after *in utero* electroporation with indicated plasmids. Golgi is determined by immunostaining with anti-GM130 antibody (red). Arrowheads point to the Golgi, arrows show radial migrating direction. Scale bar, 40 μm. **(H)** Quantification of the percentage of non-radially migrating GFP-expressing cells in upIZ and loCP regions. Histograms show mean ± SEM from at least three brains. ^∗^*p* < 0.05; ^∗∗^*p* < 0.01; ^∗∗∗^*p* < 0.001.

Next, to investigate if overexpression of *N*-cadherin could rescue the migration defect caused by loss function of Myo10, we then co-electroporated Myo10 shRNA and RFP-Ncad into the developing cortex (Supplementary Figure S1A). To our surprise, we found the migration defect caused by loss of Myo10 was rescued by overexpression of *N*-cadherin. The ratio of EGFP^+^ cells was decreased in the IZ [Myo10 shRNA: 56.71 ± 1.98%, *n* = 4; Myo10 shRNA + RFP-Ncad: 32.78 ± 7.03%, *n* = 5; *t*-test, ^∗^*p* = 0.027], and increased in the loCP compared with Myo10 shRNA [Myo10 shRNA: 12.51 ± 1.73%, *n* = 4; Myo10 shRNA + RFP-Ncad: 30.19 ± 3.99%, *n* = 5; *t*-test, ^∗∗^*p* = 0.008] (**Figures 2A,B**). Furthermore, we used time-lapse confocal microscope to visualize neuronal migration to further confirm above results. We tracked the movement of EGFP-labeled locomoting neurons and measured the speeds of these cells in development cortex. We found neurons expressing control shRNA migrated toward the upper CP at an average speed of 7.39 μm/h (**Figures 2C,D**). But knockdown of Myo10 led to decrease the migration of the locomoting neurons at an average speed of 3.43 μm/h [control shRNA: 7.39 ± 0.65, *n* = 21; Myo10 shRNA: 3.43 ± 0.89, *n* = 22; *t*-test, ^∗∗^*p* = 0.005] (**Figures 2C,D**). Overexpression of *N*-cadherin in Myo10 knocking-down neurons rescued its migration at an average speed of 7.50 μm/h, which is comparable to control level [control shRNA: 7.39 ± 0.65, *n* = 21; Myo10 shRNA + Ncad: 7.50 ± 0.83, *n* = 20; *t*-test, *p* = 0.863] (**Figures 2C,D**).

In our previous studies, we have reported that knockdown of Myo10 impaired the transition from multipolar to bipolar shape and the orientation of migration (Yu et al., 2012; Ju et al., 2013). Therefore, we wonder if *N*-cadherin can also rescue these defects. Morphological analysis revealed that overexpressing of *N*-cadherin could rescue this abnormal multipolar-bipolar transition caused by Myo10 shRNA [Myo10 shRNA: 44.33 ± 4.16%, *n* = 4; Myo10 shRNA + Ncad: 30.67 ± 2.08%, *n* = 5; *t-*test, ^∗^*p* = 0.015] (**Figures 2E,F**). After exhibiting biopolar morphologies, the neurons migrate over along the radial glial fibers to the CP. The direction of cell migration is accompanied by orientation of the Golgi apparatus localizes in front of the nucleus (Jossin and Cooper, 2011). We found that knockdown of Myo10 led to an increase of EGFP^+^ cells with non-radial migration in the upIZ and loCP regions based on GM130 staining (**Figures 2G,H**). But, overexpressing of *N*-cadherin could rescue this mis-directional migration caused by Myo10 shRNA [Myo10 shRNA: 32.67 ± 0.58%, *n* = 4; Myo10 shRNA + Ncad: 23.33 ± 0.58%, *n* = 5; *t*-test, ^∗∗∗^*p* < 0.001] (**Figures 2G,H**). Taken together, these results indicated that Myo10 regulated *N*-cadherin-mediated neuronal adherence and migration.

### Myo10 Interacts with *N*-Cadherin Cytoplasmic Domain

**FIGURE 3:**
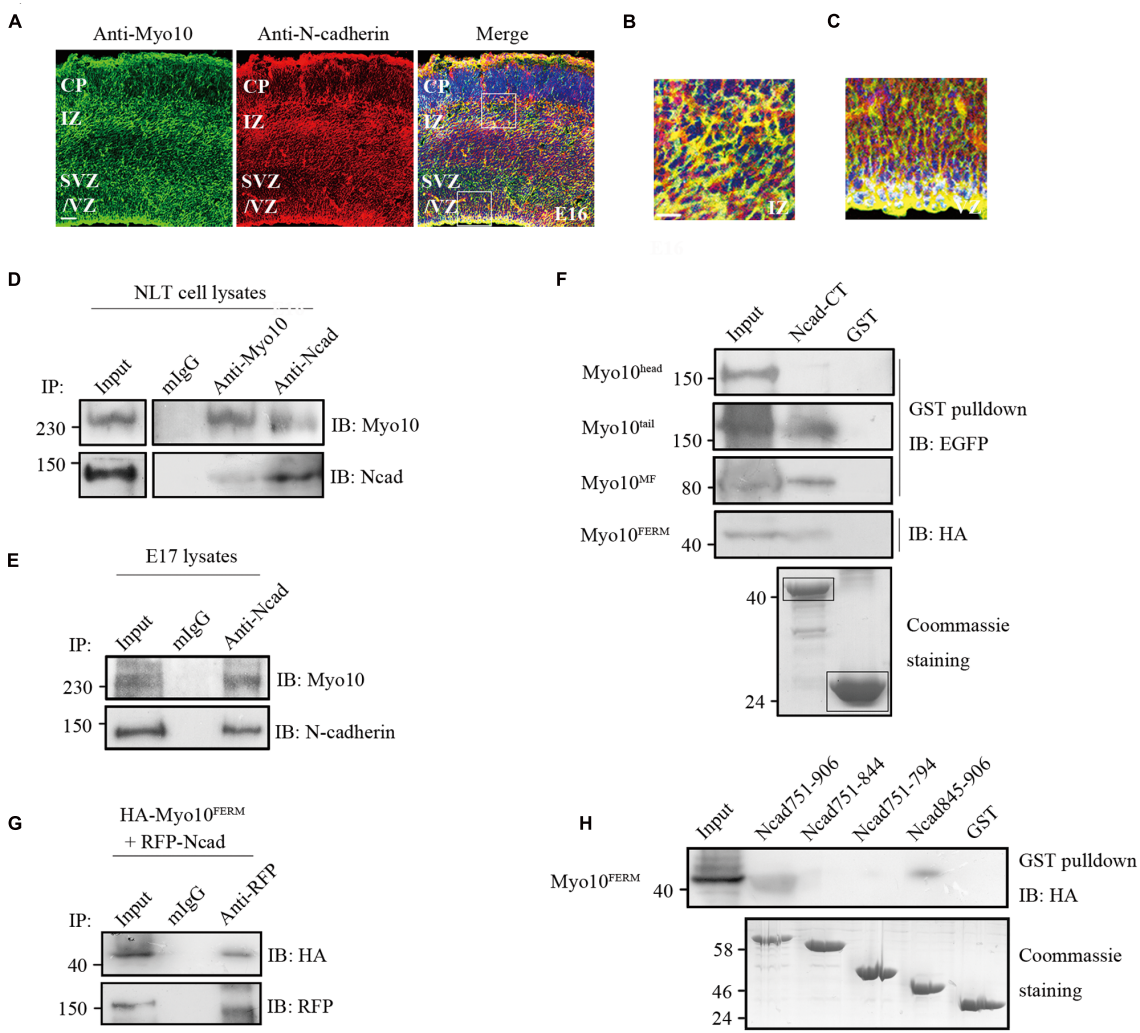
**Myo10 interacted with *N*-cadherin cytoplasmic domain. (A)** The expression of Myo10 and *N*-cadherin in E16 mouse cortex. Scale bar, 100 μm. **(B,C)** The square boxes **(A)** of IZ and VZ were magnified and were shown in **(B,C)**, respectively. Scale bar, 20 μm. **(D)** Endogenous *N*-cadherin co-immunoprecipitats with endogenous Myo10. NLT cell lysates were immunoprecipitated with anti-*N*-cadherin antibody or anti-Myo10 antibody, and with mouse IgG as control. **(E)** Coimmunoprecipitation of Myo10 with *N*-cadherin was showed in mouse brain lysate. Mouse brain lysate from E17 was incubated with anti-*N*-cadherin antibody and with mouse IgG as a control. **(F)** Immunoblotting of the pulled down fraction by GST-Ncad-CT and GSTalone. The amounts of GST-*N*-cadherin-CT and GST (indicated by boxes) were revealed by coommassie blue staining (lower panel). **(G)** Co-immunoprecipitation of *N*-cadherin and Myo10^FERM^. HA-Myo10^FERM^ was co-expressed with RFP-Ncad in HEK293T cells and immunoprecipitated by anti-RFP antibody or mIgG. **(H)** Immunoblotting of the pulled down fraction by the truncated *N*-cadherin cytoplasmic domain fused with GST and GST alone. The amounts of different GST-*N*-cadherin fusion proteins and GST were revealed by coomassie blue staining (lower panel).

Using immunohistochemical analysis, we found that both Myo10 and *N*-cadherin were co-expressed throughout the cerebral cortex at E16 when most of the neurons start to migrate, and Myo10 was co-localized with *N*-cadherin especially in the IZ and VZ (**Figures 3A–C**), which suggested Myo10 might interact with *N*-cadherin. Next, we performed immunoprecipitation assay to characterize the interaction between Myo10 and *N*-cadherin. Both Myo10 and *N*-cadherin were detected in the immunoprecipitate complexes from NLT cells (a neuronal cell line), and embryonic brain lysate (**Figures 3D,E**). Furthermore, we expressed different regions of Myo10 (Myo10^head^, Myo10^tail^, Myo10^MF^, and Myo10^FERM^) and performed a glutathione S-transferase (GST) pull-down assay to determine which region of Myo10 interacts with *N*-cadherin. Interestingly, we found the FERM domain (Myo10^tail^, Myo10^MF^, and Myo10^FERM^) were able to be pulled down by GST-N-cadherin cytoplasmic domain fusion protein (GST-Ncad-CT), whereas Myo10^head^ without the FERM domain abolished such interaction (**Figure 3F**). We also found expression of Myo10^FERM^ was able to pull-down of RFP-Ncad (**Figure 3G**). To further identify the Myo10-binding region within the *N*-cadherin, we expressed truncated *N*-cadherin cytoplasmic domain fused with GST (GST-Ncad751-906, GST-Ncad751-844, GST-Ncad751-794, and GST-Ncad845-906) and perform GST pull-down assay. We found deletion of the amino acids 845–906 within the *N*-cadherin cytoplasmic tail led to disruption of the *N*-cadherin-Myo10^FERM^ interaction (**Figure 3H**). These data suggested that Myo10 interacted with the intracellular domain of *N*-cadherin through its FERM domain.

### Myo10 is Involved in Maintenance of Cell Surface *N*-Cadherin

**FIGURE 4:**
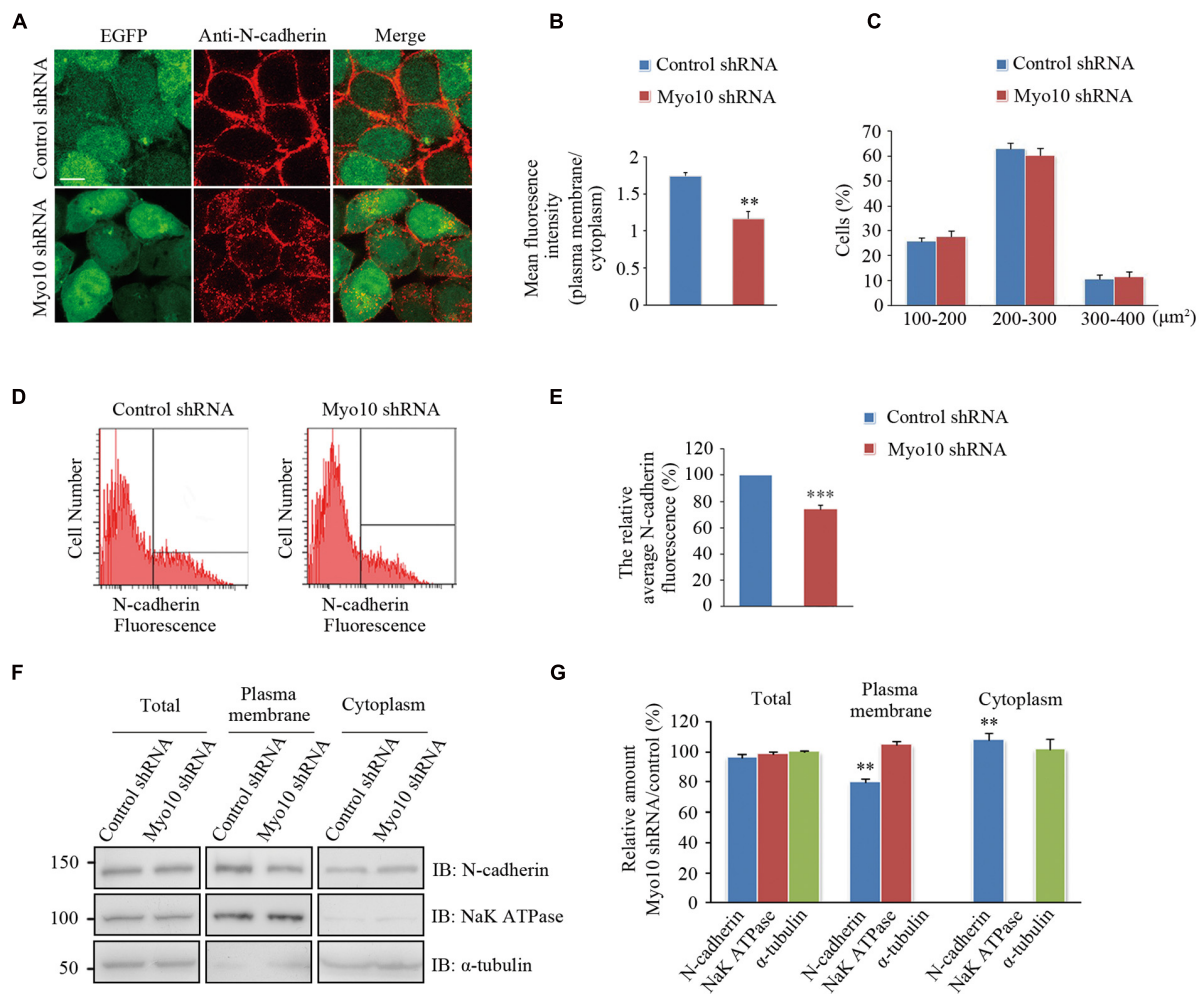
**Knockdown of Myo10 perturbed the distribution of *N*-cadherin. (A)** HEK 293T cells expressing control shRNA or Myo10 shRNA (green) were immunostained with anti-*N*-cadherin antibody (red). Scale bars: 10 μm. **(B)** Quantification of the ratio of *N*-cadherin fluorescence intensity of the plasma membrane region to that of cytoplasmic region. Histograms show mean ± SEM from at least 30 cells. **(C)** Quantification of the percentages of cells in 100–200 μm^2^, 200–300 μm^2^, 300–400 μm^2^, respectively. Histograms show mean ± SEM from at least three independent experiments. **(D)** FACS analysis of HEK 293T cells transfected with the indicated plasmids. *Y* axes and *X* axes indicate the cell number and the fluorescence intensity of *N*-cadherin, respectively. **(E)** Quantification of the relative cell surface *N*-cadherin fluorescence. Histograms show mean ± SEM from at least three independent experiments. **(F)** Western blot analysis of the total, the plasma membrane and the cytoplasm proteins of HEK 293T cells transfected with the indicated plasmids. **(G)** Quantification of the relative amounts in Myo10-knockdown versus control cells. Histograms show mean ± SEM from at least three independent experiments. ^∗∗^*p* < 0.01; ^∗∗∗^*p* < 0.001.

We next investigated the effects of Myo10-mediated *N*-cadherin subcellular distribution. An expression construct encoding a short hairpin RNA (shRNA) which has been verified in our previous studies (Zhu et al., 2007; Yu et al., 2012). We found knockdown of Myo10 led to an impairment of the *N*-cadherin distribution by reducing it from the cell periphery and increasing in the cytoplasm [Mean fluorescence intensity of plasma membrane to cytoplasm in control cells: 1.74 ± 0.05, *n* = 31; Myo10-knockdown cells: 1.16 ± 0.09, *n* = 33; *t*-test, ^∗∗^*p* = 0.003] (**Figures 4A,B**). Comparing to control cells, the morphology of cell with Myo10 knockdown was not affected when we measured the cell total area (**Figure 4C**). Using FACS to analyze the surface *N*-cadherin expression, the fluorescent intensity of *N*-cadherin was decreased ∼25% comparing to control cells [The ratio of surface *N*-cadherin in Myo10-knockdown cells to that of control was 74.25 ± 3.19%, *n* = 3; *t*-test, ^∗∗∗^*p* < 0.001] (**Figures 4D,E**). Through isolating plasma membrane and cytoplasm fraction, we further confirmed that knockdown of Myo10 led to a decrease of the amount of *N*-cadherin in the plasma membrane [79.88 ± 1.39%, n = 3; *t*-test, ^∗∗^*p* = 0.002], but an increase of the cytoplasmic *N*-cadherin [112.7 ± 5.71%, *n* = 3; *t*-test, ^∗∗^*p* = 0.009] (**Figures 4F,G**). However, no apparent change of the total level of *N*-cadherin was observed in the Myo10-knockdown cells [96.49 ± 1.99%, *n* = 3; *t*-test, *p* = 0.093] (**Figures 4F,G**). Furthermore, using immunohistochemical analysis of brain section, we found expression of Myo10 shRNA promoted the intracellular accumulation of endogenous *N*-cadherin (Supplementary Figure S2). Therefore, the above data suggested Myo10 might regulate surface *N*-cadherin expression through directly modulating its cytoplasm-to-membrane transportation.

### Myo10 Regulates the Membrane Trafficking of *N*-Cadherin to Maintain the Surface *N*-Cadherin Expression

**FIGURE 5:**
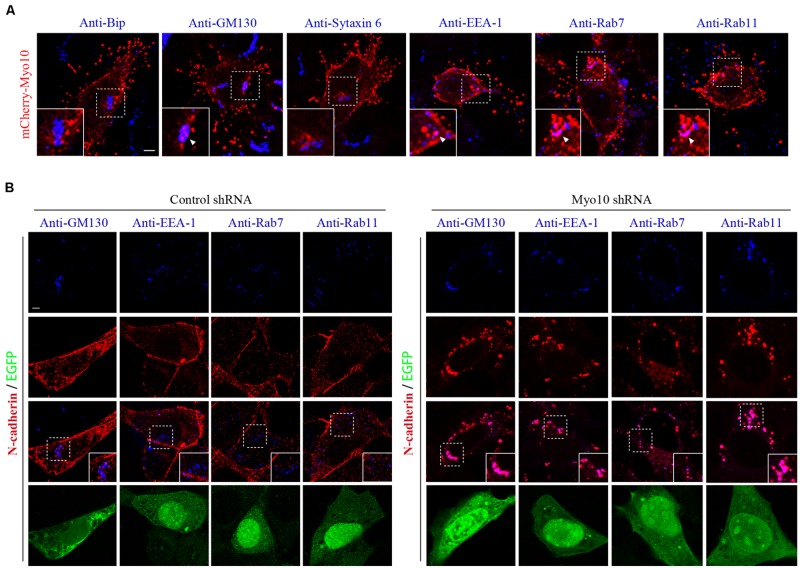
**Knockdown of Myo10 resulted in localization of *N*-cadherin into Golgi apparatus and endocytic vesicles. (A)** NLT cells expressing mCherry-Myo10 (red) were immunostained with endosomal markers: Bip, GM130, Sytaxin6, EEA-1, Rab7 and Rab11 (blue). Scale bar, 5 μm. **(B)** NLT cells expressing control shRNA or Myo10 shRNA (green) were immunostained with anti-*N*-cadherin antibody (red) and endosomal markers (blue): GM130, EEA1, Rab7 and Rab11. Scale bar, 5 μm.

The regulation of cadherins by membrane trafficking is emerging as a key player in cell adherence. Previous studies demonstrated that Myo10 potentially transported or localized proteins by protein-protein interactions through its FERM domain (Tokuo and Ikebe, 2004; Zhang et al., 2004; Pi et al., 2007; Zhu et al., 2007; Almagro et al., 2010). Next, we investigated whether Myo10 regulated cellular distribution of *N*-cadherin. Using *N*-cadherin immunostaining, we found knockdown of Myo10 led to abolished *N*-cadherin expression in cell periphery, but mainly localized its expression to the cytoplasm (**Figures 4A** and **5B**). To determine whether there were Myo10 positive vesicles within the cells, we transfected cells with mCherry-Myo10 plasmid and performed immunostaining with a series of vesicle markers, including Bip (a marker of endoplasmic reticulum), GM130 (a marker of Cis-Golgi membrane), Sytaxin6 (a marker of trans-Golgi membrane), EEA-1 (a marker of early endosome), Rab7 (a marker of late endosome) and Rab11 (a marker of recycling endosome). We found Myo10-containing vesicles were negative for Bip, Sytaxin6, while were partially overlapped with GM130, EEA-1, Rab7 and Rab11 (**Figure 5A**). Next, we found knockdown of Myo10 led to increased colacolization of *N*-cadherin with GM130, which suggested that Myo10 might accompany *N*-cadherin as it is transported from Golgi to the plasma membrane. Furthermore, knowdown of Myo10 resulted in *N*-cadherin overlapping with EEA1 and Rab11 positive vesicles (**Figure 5B**), which suggested that Myo10 also regulated endosomal sorting of *N*-cadherin containing vesicles. Therefore, the above data suggested that Myo10 governed cell surface *N*-cadherin expression through regulating its membrane trafficking.

## Discussion

In this study, we provided evidence that Myo10 interacted with *N*-cadherin through its FERM domain and thus participated in *N*-cadherin mediated cell to cell adhesion and neuronal migration. Furthermore, we found Myo10 regulated *N*-cadherin surface expression through controlling it membrane trafficking. Taken together, these results illustrated the critical role of Myo10 in the *N*-cadherin-mediated neuronal migration during brain development.

Myo10 acts as an actin-based motor protein to induce filopodia with its striking localization at the tips of filopodia (Berg and Cheney, 2002; Bohil et al., 2006; Watanabe et al., 2010), a polarized structures at migrating cell leading edge. The formation of filopodia requires PtdIns(3,4,5)P3 that localizes to the leading edge of migrating cells and binds with the Myo10-PH2 domain for regulation of filopodia dynamics. Given the importance of filopodia in neuronal development, particularly in axon outgrowth and branching (Gallo and Letourneau, 1998; Luo, 2002; Dent et al., 2007; Kwiatkowski et al., 2007), Myo10 also played critical roles in neuronal development (Sousa et al., 2006; Zhu et al., 2007; Nie et al., 2009; Raines et al., 2012). Our previous studies have explored the importance of Myo10 in neuronal radial migration. Suppression of Myo10 via *in utero* electroporation resulted in a significant reduction of migrating neurons in CP and most of neurons stored in IZ, which suggested that Myo10 was also required for cortical neuron migration. Several factors may account for Myo10-deficientneuronal migration defects, including the Myo10 functions in membrane transport or actin filament organization. Previously, we have reported that Myo10 interacted with DCC, a receptor of Netrin-1 which is involved in axon guidance, and this interaction was required for radial orientation of the migrating neurons (Zhu et al., 2007; Ju et al., 2013). In this study, we showed Myo10-knockdown neurons displayed an abnormal migration process, and these cells did not attach tightly to the radial glial fibers when glial-guided migration was ongoing. Importantly, *N*-cadherin is expressed in both neuron and glial cells and plays a role in the locomotion mode of migration along radial glial fibers (Jossin and Cooper, 2011). Surprisingly, by coating cell culture plates with NcadECD-Fc, we found that knockdown of Myo10 in neurons phenocopied the effects of *N*-cadherin in binding to NcadECD-Fc, which indicated that Myo10 was required for *N*-cadherin-mediated cell adhesion. Therefore, these observations suggested that Myo10 was not only involved in the critical transition from the multipolar to bipolar stage during the neuronal migration, but also engaged in the essential phase of glial-guided migration for the development of the cerebral cortex. Our findings support and extend the previous studies that indicated the potential function of Myo10 in cortex development.

Myosins constitute a superfamily of motor proteins that play important roles in cellular and organismal physiology. Previously, myosin superfamily – myosin II, VI, and VII – have been implicated in cadherin-mediated cell adherence. Conventional non-muscle myosin II can be recruited and activated in response to *E*-cadherin ligation, where it serves to promote adhesion and the local accumulation of cadherin at cell–cell contacts. Myosin VII was reported to associate indirectly with *E*-cadherin through the transmembrane protein vezatin. Myosin VI, an unconventional myosin motor, can be also recruited to *E*-cadherin adhesion at a late stage in the maturation of cultured epithelial monolayers. Nonetheless, as an unconventional myosin, Myo10 has been known to play a prominent role in extracellular adhesion by transporting intergrins or VE-cadherin along filopodia through its FERM domain (Zhang et al., 2004; Almagro et al., 2010). However, little is known about its function in the regulation of cell-cell adherence. Recently, Myo10 has been known to require for the *E*-cadherin-mediated assembly of cell–cell contacts (Liu et al., 2012). In our study, we found that in the embryonic brain Myo10 and *N*-cadherin existed in a protein complex. Furthermore, Myo10 interacted with cytoplasmic domain of *N*-cadherin through its FERM domain and regulated *N*-cadherin-mediated cell–cell adherence. Our novel discovery supports the versatility of Myo10 for interacting with different adhesive receptors including *N*-cadherin and reveals an important contribution of Myosins family to cadherin function.

As an unconventional motor protein, Myo10 has been reported to act as a cargo carrier for membrane protein transport along actin cables. *N*-cadherin has been known to traffic between membrane and cytoplasm to execute its biological function, which is regulated through Rab GTPase-dependent endocytic pathways (Kawauchi et al., 2010) and Reelin/Rap1 signaling (Franco et al., 2011; Jossin and Cooper, 2011). In our study, we found suppression of Myo10 perturbed the subcellular distribution of *N*-cadherin by abolishing the surface level of *N*-cadherin, which suggested that *N*-cadherin could be delivered to the membrane with the aid of Myo10. So next we performed immunofluoresence assays and found that Myo10 was associated with GM130 positive Golgi apparatus membrane and endocytic vesicles, such as EEA1, Rab7 and Rab11 positive vesicles. Knockdown of Myo10 led to stalled *N*-cadherin within Golgi apparatus and EEA1 and Rab11 positive endocytic vesicle in the cytoplasm, but barely within Rab7 positive late endosome, which suggested that Myo10 may not only regulate transporting of *N*-cadherin from Golgi apparatus to the membrane, but also control the endosomal sorting of *N*-cadherin containing vesicles. Interestingly, we found overexpression of *N*-cadherin could overcome the defects of cortical neuronal migration caused by knockdown of Myo10. Therefore, we speculated that oversaturation of *N*-cadherin in the endosomal sorting pathway may compromise its membrane expression caused by Myo10 knockdown. Furthermore, we found that overexpression of *N*-cadherin indeed has normal membrane *N*-cadherin expression in Myo10 knockdown cells (Supplementary Figures S1A,B). Thus, it is not surprising that an overexpression of *N*-Cadherin can rescue the Myo10-knockdown phenotype. Besides, the recent studies (Kawauchi et al., 2010; Jossin and Cooper, 2011) suggest that Rap1 and Rab5/Rab11 are involved in *N*-cadherin distribution, then affect cell adhesion during the neuronal migration. Therefore, our data revealed a novel mechanism of Myo10 interacting with *N*-cadherin and regulating its cell-surface expression through participating in vesicle trafficking, which may explain that loss of Myo10 results in the defects of *N*-cadherin-mediated neuronal adhesion and migration during brain development.

## Conclusion

Our data suggest a model whereby Myo10 participates in *N*-cadherin function and thereby regulates cortical neuronal migration. Myo10 forms a complex with *N*-cadherin and then participates in the membrane trafficking of *N*-cadherin to regulate the cell surface expression, which contributes to maintain the neuronal adhesion during neuronal radial migration. However, we cannot completely rule out the possibility that Myo10 regulates the subcellular distribution of *N*-cadherin via other pathways. Indeed, the intracellular transport of *N*-cadherin depends on the vesicle traffic and assembling of the cytoskeleton is crucial for the functional protein distribution. Thus, future studies should address the detailed molecular mechanisms through which Myo10 regulates these processes.

## Author Contributions

The overall study was conceived and supervised by XZ and WG. ML and YG designed, performed and analyzed most of the experiments. JM modified the immunofluorescence. HY performed the experiments with dissociated neurons. DZ performed the stainings on frozen sections. WF supported the western-blotting. XJ supported the *in vivo* experiments. MS and YM contributed in materials. WX provided the Myo10 plasmids and antibody. ML and XZ wrote the manuscript.

## Conflict of Interest Statement

The authors declare that the research was conducted in the absence of any commercial or financial relationships that could be construed as a potential conflict of interest.
