# Supplementary material for: Myosin X regulates neuronal radial migration through interacting with N-cadherin
Source: Front Cell Neurosci. 2015 Aug 18;9:326. doi: 10.3389/fncel.2015.00326 (PMC4539528; doi:10.3389/fncel.2015.00326)
Supplement: Supplementary file 1 [file Image_1.PDF]

Supplementary Figure 1

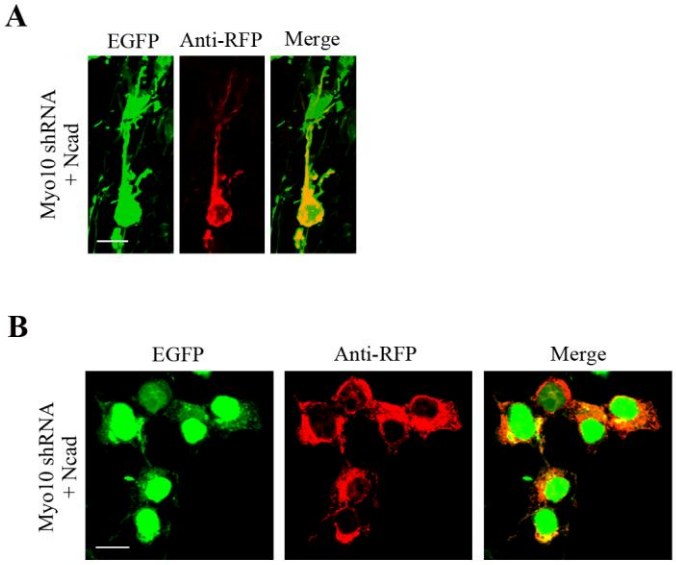

Supplementary Figure 1 (A) E18.5 cerebral cortices were electroporated with indicated plasmids at E15.5. Frozen sections were immunostained with anti-EGFP (green) and anti-RFP (red) antibodies to detect the expression of RFP-tagged N-cadherin in migrating neurons. Scale bar, 10 μm. **(B)** HEK 293T cells co-expressing Myo10 shRNA (green) and RFP-Ncad were immunostained with anti-RFP antibody (red). Scale bars: 10 μm.
