# Supplementary material for: Myosin X regulates neuronal radial migration through interacting with N-cadherin
Source: Front Cell Neurosci. 2015 Aug 18;9:326. doi: 10.3389/fncel.2015.00326 (PMC4539528; doi:10.3389/fncel.2015.00326)
Supplement: Supplementary file 2 [file Image_2.PDF]

Supplementary Figure 2

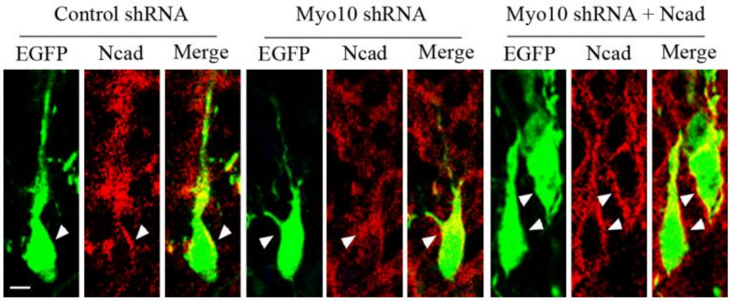

Supplementary Figure 2 E18.5 cerebral cortices were electroporated with indicated plasmids at E15.5. Frozen sections were immunostained with anti-EGFP (green) and anti-N-cadherin (red) antibodies to detect the expression of endogenous N-cadherin in migrating neurons. Scale bar, 5 μm.
